# Peripheral primitive neuroectodermal tumor: Dynamic CT, MRI and clinicopathological characteristics - analysis of 36 cases and review of the literature

**DOI:** 10.18632/oncotarget.2649

**Published:** 2014-12-15

**Authors:** Yan Tan, Hui Zhang, Guo-lin Ma, En-hua Xiao, Xiao-chun Wang

**Affiliations:** ^1^ Department of Radiology, First Clinical Medical College, Shanxi Medical University, Taiyuan 030001, Shanxi Province, China; ^2^ Department of Radiology, China-Japan Friendship Hospital, Beijing 100029, China; ^3^ Department of Radiology, Second Xiangya Hospital, Central South University, Changsha 410011, Hunan Province, China

**Keywords:** pPNET, CT, MRI, clinicopathology

## Abstract

**Background:**

The peripheral primitive neuroectodermal tumor (pPNET) is a rare malignant tumor originating from neuroectoderm. The accurate diagnosis is essential for the treatment of pPNET.

**Methods:**

we performed the largest cases of retrospective analysis thus far to review the unique computed tomography (CT), magnetic resonance imaging (MRI), and clinicopathological features of pPNET. The tumor location, morphological features, signal intensity, contrast enhancement characteristics, and involvement of local soft tissues of 36 pPNETs were assessed.

**Results:**

Our results showed that there were more men (25/36) than women pPNETs patients. Unenhanced MRI (16 cases) showed that 14 cases were isointense and 2 cases were hypointense on T1WI. Nine cases were isointense and 7 were hyperintense on T2WI. Most pPNETs had heterogeneous signal intensity with small necrosis (CT: 31/36; MRI: 14/16) as well as heterogeneous enhancement (CT: 34/30; MRI: 15/16). The tumors usually had ill-defined borders and irregular shapes (CT: 30/36; MRI: 15/16). Pathologic exam showed small areas of necrosis in all tumors.

**Conclusions:**

The diagnosis of pPNET should be suggested in young men when the imaging depicts a single large ill-defined solid mass with small area of necrosis, especially for those whose images show iso-intense on T1WI and T2WI and have heterogeneous enhancement.

## INTRODUCTION

The term primitive neuroectodermal tumor (PNET) was coined by Hart and Earle in 1973 [1] to describe a rare malignant tumor originating from neuroectoderm [2]. The incidence of peripheral primitive neuroectodermal tumor (pPNET) is low, accounting for only about 4% of all soft tissue tumors [3]. It was reported that CD99 is highly expressed in pPNET with the characteristics of chromosomal translocation t(11;22)(q24;q12) [4]. pPNET is a clinically aggressive tumor and has gloomy prognosis. Due to their rarity, the radiologic diagnosis of pPNET can be challenging. Compared with pathological diagnosis, the CT and MRI could noninvasively describe the tumor location, morphology, size, margin, blood supply or even the pathological nature before the surgery. Only a few reports describing the computed tomography (CT) and magnetic resonance imaging (MRI) features of pPNETs have been published [5–7]. Although there are some common features in the CT, MRI, and clinicopathologic appearances between the pPNET and other malignant tumors, the analysis of characteristic imaging and clinical features of pPNET could lead to a specific diagnosis.

In this study, we performed the retrospective analysis of CT, MRI, and clinicopathological characteristics of 36 pPNETs diagnosed by pathology. Our results suggested that pPNET has unique imaging and clinical features which can be used for the diagnosis.

## RESULTS

### Clinical data

The clinical findings from the 36 cases are summarized in Table 1. Patient age ranges from 1 to 66-year-old (The average is 30-year-old). We found that patients with pPNET arising in the bone were younger than those arising in the soft tissue and organ (*p* < 0.05). There were more male patients (25/36) than females (11/36), especially in the bone group (15/4, male/female). Twenty-nine patients presented with intermittent pain, 4 with cough, 1 as an incidental finding on physical examination and 2 with dizziness and fatigue. Out of 36 patients, 25 (69.4%) had 12 month postoperative follow-up, 12 patients died of which 9 had metastases and 3 had complications, 9 patients recurred of which 3 had metastases and 6 local recurrence, and 4 had no recurrence. pPNET patients arising from organs had a much poorer prognosis than those tumors arising in bone or soft tissues.

**Table 1:** Clinical findings in patients with pPNET

Tumor type	Cases	% with pPNET	Mean age (years)	Gender (M/F)	Treatment	Follow up
Bone	19	53%	23.3	15/4	Tumor resection 19 Chemotherapy 14	13 cases Died 6 Recurred 4 Remission 3
Soft tissue	10	28%	32.9	5/5	Tumor resection 9 Chemotherapy 5 Radiotherapy 1	7 cases Died 2 Recurred 4 Remission 1
Organ	7	19%	44.4	5/2	Tumor resection 5 Chemotherapy 3	5 cases Died 4 Recurred 1 Remission 0
Total	36	-	30.0	25/11	-	25 cases Died 12 Recurred 9 Remission 4

pPNET: peripheral primitive neuroectodermal tumor; The time of follow up was 12 months after surgery.

### Dynamic CT and MRI findings

Dynamic CT and MRI findings from 36 patients were summarized in Table 2 and Table 3, respectively. CT and MRI imaging showed a single large soft tissues mass with a mean diameter of 8.1 cm (range: 1.8–27 cm). Tumors were identified in bone (*n* = 19: limbs 11, vertebrae 4, craniofacial skeleton 4), soft tissues (*n* = 10: pelvis 2, retroperitoneum 2, groin 1, nasopharynx 1, chest wall 2, mediastinum 2), and organs (*n* = 7: parotid 1, lung 4, spleen 1, liver 1).

**Table 2:** The Dynamic CT findings with pPNET

Tumor type	Cases	Location	Mean size (cm)	Density	Uneven	Border (ill-defined)	Enhanced	Invasion	Lymph node metastasis
Bone	19	Limbs(11) Vertebral(4) Craniofacial skeleton (4)	8.9	HOD: 4 ID: 15 HED: 0	16	17	18 MI: 1 MO: 4 SI: 13	16	no
Soft tissue	10	Pelvis (2) Retroperitoneum (2) Groin (1) Nasopharynx (1) Chest wall (2) Mediastinal (2)	7.4	HOD: 7 ID: 3 HED: 0	9	8	9 MI: 2 MO: 0 SI: 7	8	1 (groin)
Organ	7	Parotid (1) Pulmonary (4) Spleen (1) Liver (1)	7.1	HOD: 5 ID: 2 HED: 0	6	5	7 MI: 1 MO: 1 SI: 5	5	1 (liver) 1 (Spleen)
Total	36	-	8.1	HOD: 16 ID: 20 HED: 0	31	30	34 MI: 4 MO: 5 SI: 25	29	3

HOD: hypo-density; II: iso-density; HED: hyper-density;

MI: mild enhancement; MO: moderate enhancement; SI: significant enhancement.

**Table 3:** The Dynamic MRI findings with pPNET

Tumor type	Cases	Location	Mean size (cm)	T1WI	T2WI	uneven	Border (ill-defined)	Enhanced	Invasion	Lymph node metastasis
Bone	10	Limbs(6) Vertebral(3) Craniofacial skeleton (1)	8.2	II: 9 HOI:1 HEI: 0	II: 6 HOI: 0 HEI: 4	9	9	9 MI: 1 MO: 0 SI: 8	8	no
Soft tissue	6	Pelvis (2) Retroperitoneum (2) Nasopharynx (1) Mediastinal (1)	5.7	II: 5 HOI: 1 HEI: 0	II: 3 HOI: 0 HEI: 3	5	6	6 MI: 0 MO: 0 SI: 6	6	no
Total	16	-	7.3	II: 14 HOI: 2 HEI: 0	II: 9 HOI: 0 HEI: 7	14	15	15 MI: 1 MO: 0 SI: 14	14	no

HOI: hypo-intense; II: iso-intense; HEI: hyper-intense;

MI: mild enhancement; MO: moderate enhancement; SI: significant enhancement.

Unenhanced CT (36 cases) revealed that solid tumors were either isodense (*n* = 20) or hypodense (*n* = 16). Unenhanced MRI (16 cases) showed that 14 cases were isointense and 2 cases were hypointense on T1WI. Nine cases were isointense and 7 were hyperintense on T2WI. (Figure. 1–8)

**Figure 1:**
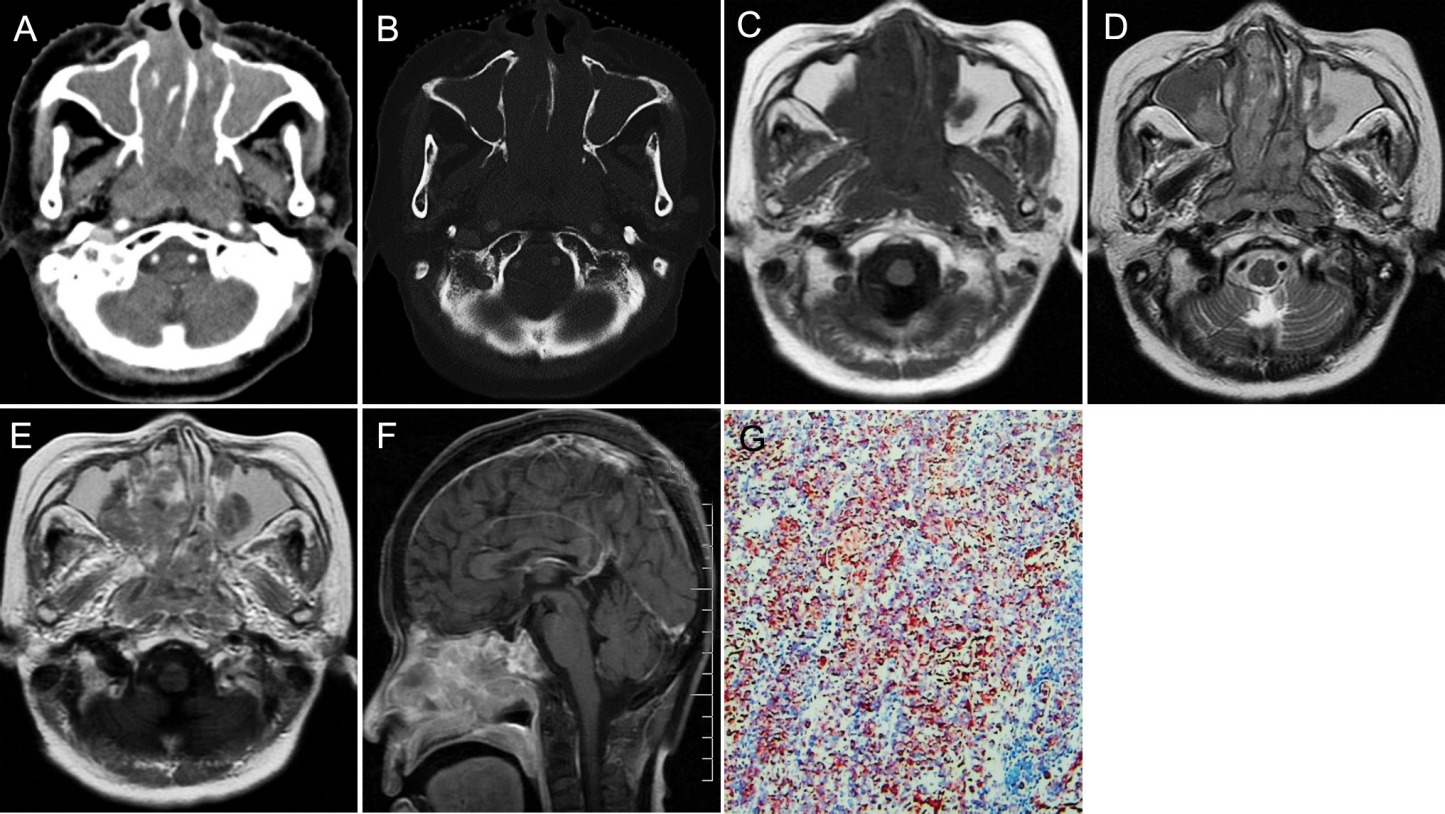
pPNET in a 63-year-old man CT imaging showed an irregular nasopharynx mass with ill-defined borders. The mass had uneven enhancement **(A)**. The sphenoid pterygoid demonstrated bone resorption **(B)**. Precontrast MRI showed a hypo-intense mass on T1WI **(C)** and hyper-intense mass on T2WI **(D)**. Enhanced MRI showed significant and heterogeneous enhancement **(E)**. Sagittal enhanced MRI **(F)** images showed the mass invaded the slopes and anterior cranial fossa. The small round tumor cells were positive for CD-99 (G × 100).

**Figure 2:**
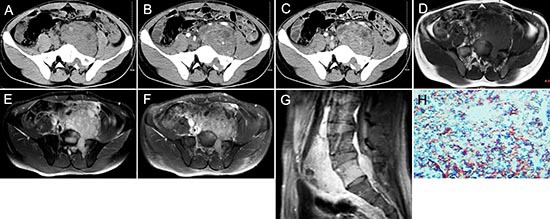
pPNET in a 21-year-old man Precontrast CT images showed a lobular iso-dense mass with necrosis in the left retroperitoneum **(A)**. Enhanced CT images showed the mass had heterogeneous contrast uptake **(B-C)**. Precontrast MRI showed the mass had an ill-defined border that was iso-intense on T1WI **(D)** and hyper-intense on T2WI **(E)**. Contrast MRI showed the mass had significant and heterogeneous enhancement **(F)**. Sagittal enhanced MRI images showed the mass invaded the 5th lumbar vertebrae and spinal canal **(G)**. The small round tumor cells were positive for CD-99 (H × 100).

**Figure 3:**
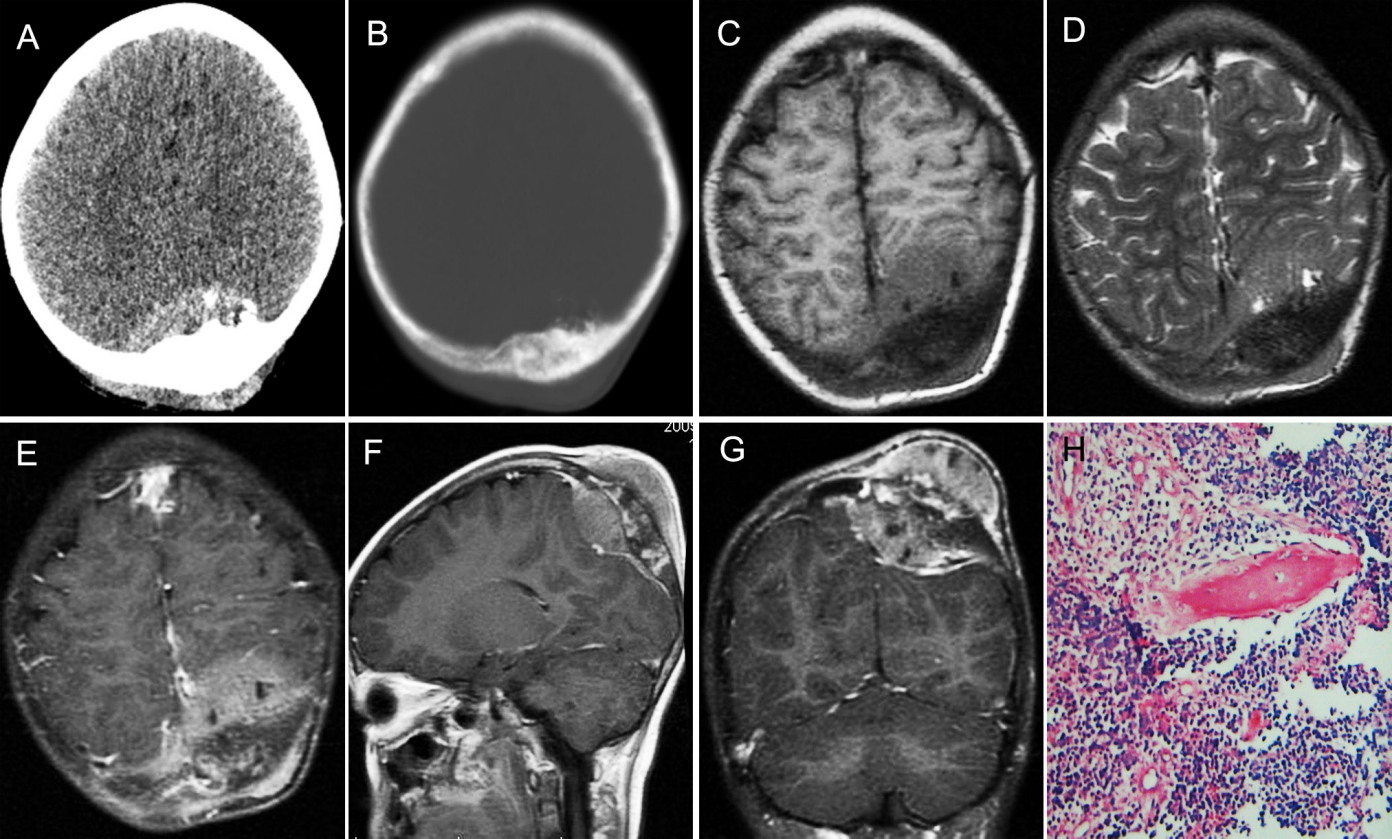
pPNET in an 11-year-old girl Enhanced CT images showed the left parietal bone had expansive destruction and a fusiform soft tissue mass with enhancement **(A and B)**. Precontrast MRI showed the mass was iso-intense on T1WI **(C)** and iso-intense on T2WI **(D)**. Contrast MRI showed the mass had significant and heterogeneous enhancement **(E)**. Sagittal **(F)** and coronal **(G)** enhanced MRI images showed dural invasion. H&E staining (H × 100) revealed that the tumor tissue consisted of poorly differentiated small round cells.

**Figure 4:**
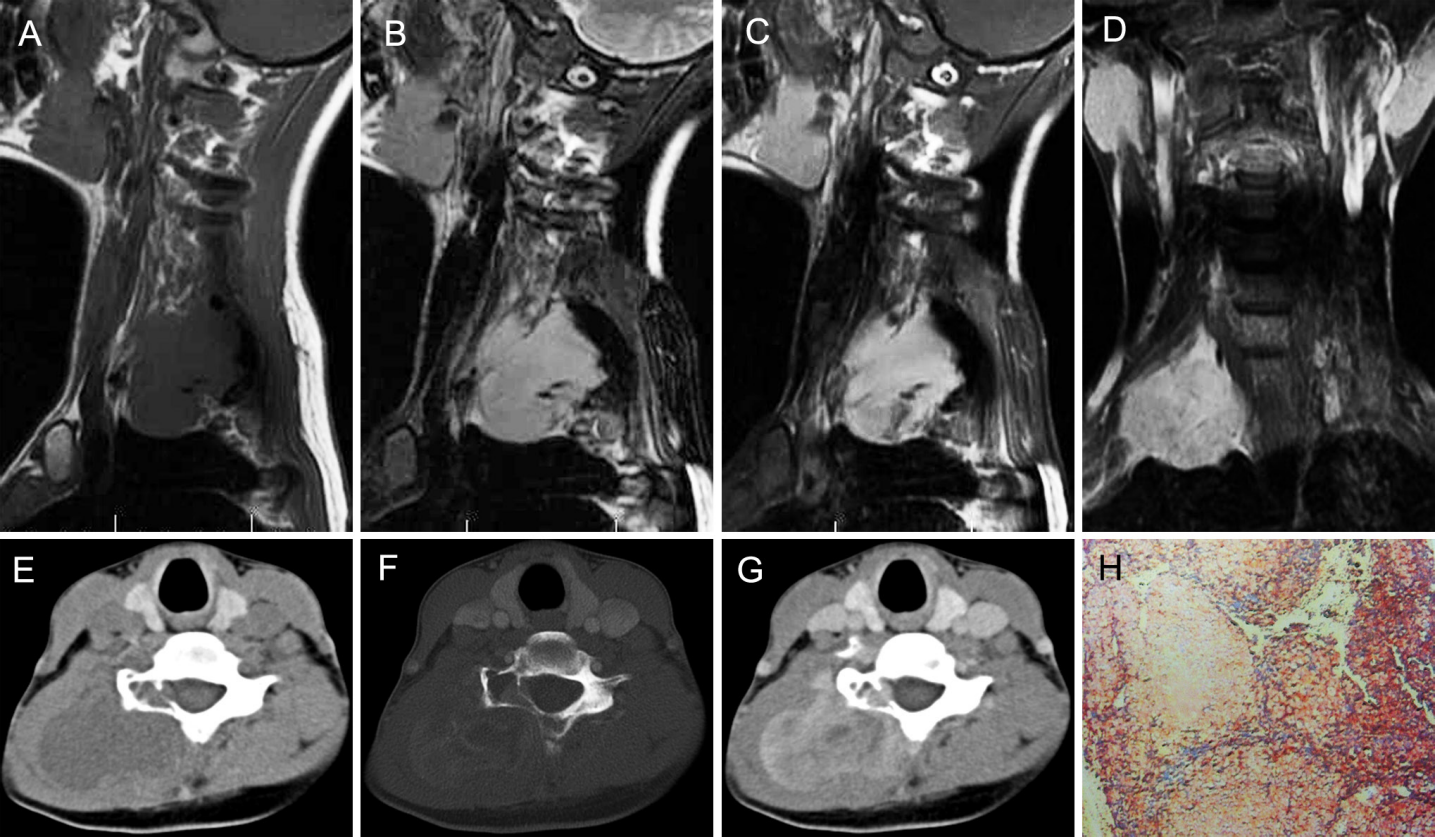
pPNET in a 20-year-old woman Precontrast MRI showed a lobulated soft tissue mass in the right neck that was iso-intense on T1WI **(A)** and hyper-intense on T2WI **(B)**. Contrast MRI showed the mass had significant and heterogeneous enhancement **(C)**. Enhanced coronal MRI **(D)** showed a mass with poorly defined margins. Precontrast CT images showed a large low-density mass with a well demarcated margin **(E)**. Bone windows showed destruction of the right vertebrae **(F)**. Contrast CT images showed the mass had slight heterogeneous enhancement **(G)**. The poorly differentiated small round tumor cells were CD99 positive (H × 100).

**Figure 5:**
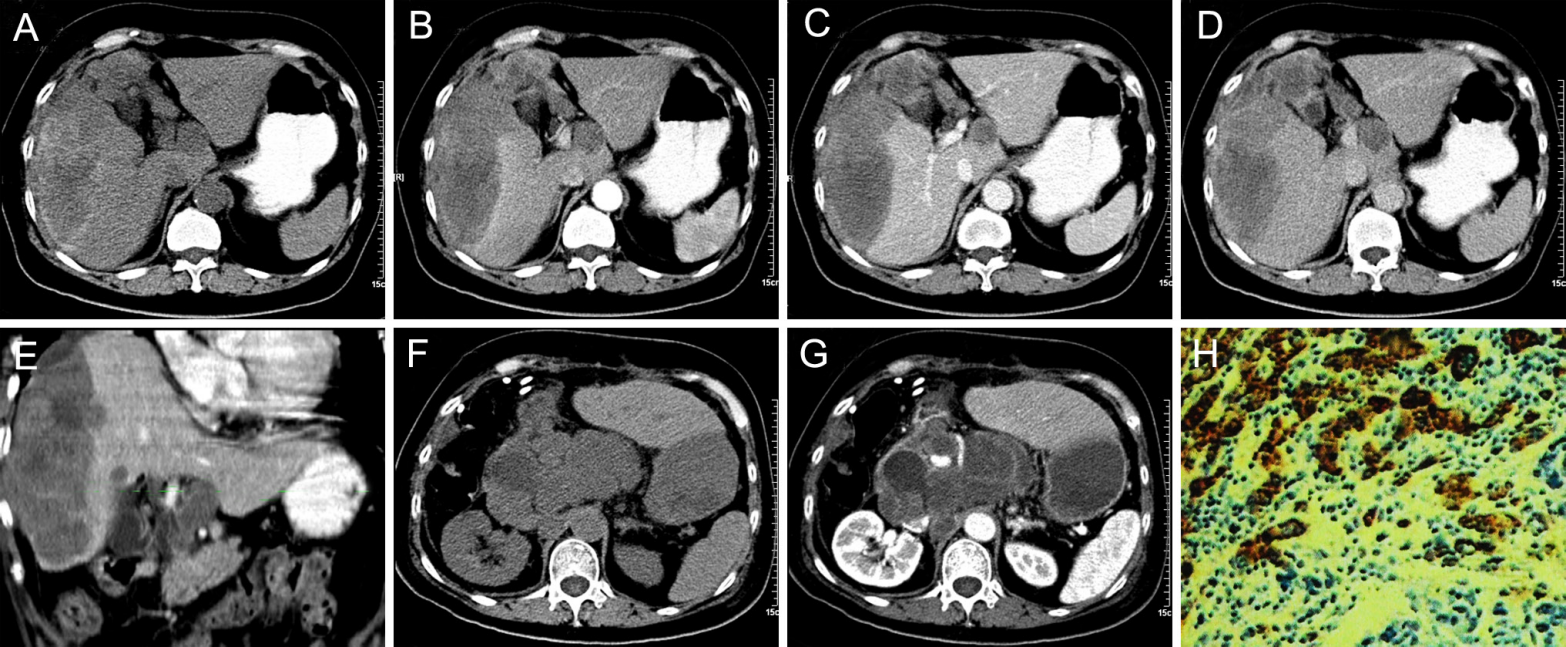
pPNET in a 66-year-old woman Precontrast CT images showed an irregular heterogeneous low-density mass with hemorrhage and ill-defined margins in the right lobe of the liver **(A)**. The enhanced CT scan showed the mass had slight ring enhancement **(B-D)**. Coronal reconstruction of the enhanced CT images showed the mass invaded the hepatic portal vein **(E)**. The patient had recurrence and retroperitoneal lymph node metastases one month after surgery **(F and G)**. The poorly differentiated small round tumor cells were CD99 positive (H × 100).

Most pPNET had heterogeneous intensity with small necrosis (CT: 31/36; MRI: 14/16) (Figure 2 and Figure 3). Only one case located in soft tissues had a specific classification and three cases located in soft tissue (Figure 2) and liver (Figure 5) had a little hemorrhage. The tumors usually had ill-defined borders and irregular shapes (CT: 30/36; MRI: 15/16) (Figure 1). Most tumors (CT: 34/36, MRI: 15/16) had heterogeneous enhancement (Figure 4), of which 25 cases on CT and 14 cases on MRI had significant enhancement. Three patients had lymph node metastases (Figure 5) and 29 had invasion into adjacent organs. No patient had distant metastases.

### Gross pathology and immunohistochemistry findings

**Figure 6:**
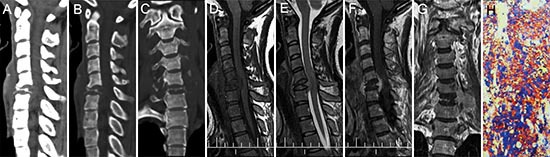
pPNET in a 26-year-old woman CT imaging showed compression fractures in the 6th cervical vertebra **(A-C)**. Precontrast MRI showed an irregular mass around the compressed 6th cervical vertebra that was iso-intense on T1WI **(D)** and iso-intense on T2WI **(E)**. Contrast MRI showed the mass had significant enhancement **(F)**. Coronal images of the enhanced MRI showed invasion into the paraspinal soft tissues **(G)**. The poorly differentiated small round tumor cells were CD99 positive (H × 100).

**Figure 7:**
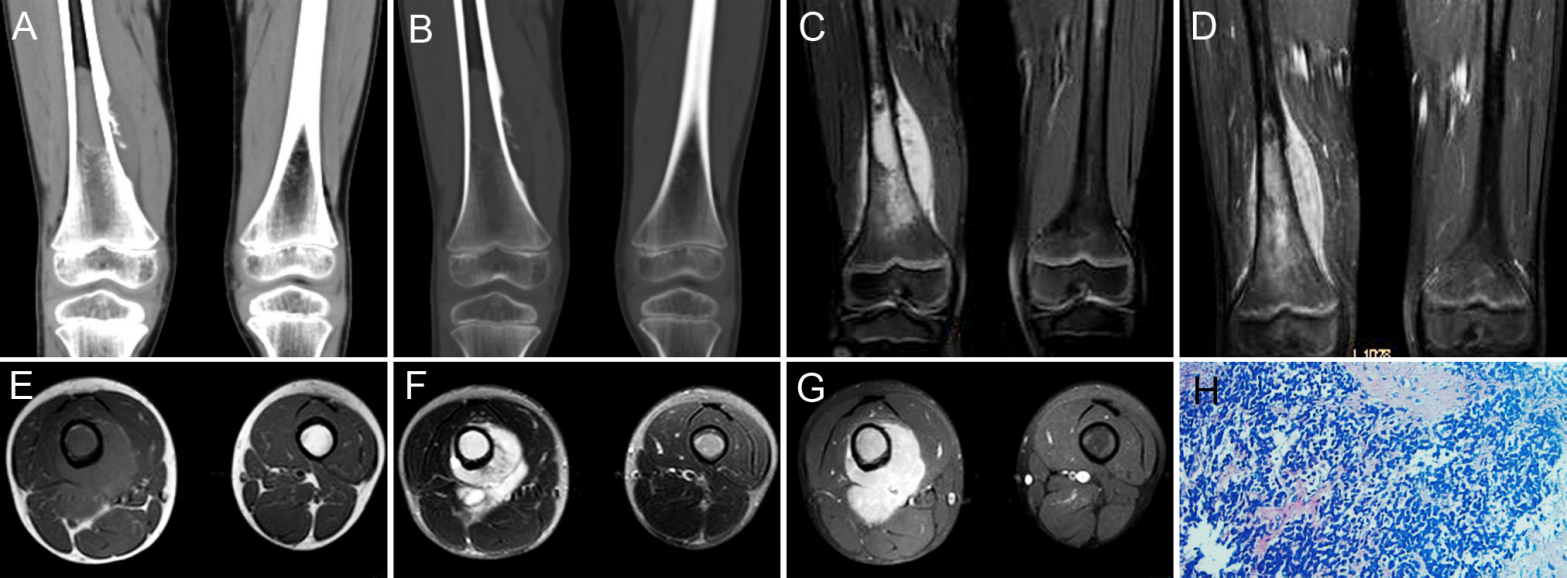
pPNET in a 9-year-old boy Precontrast CT images showed bony destruction in the right distal femur with irregular iso-dense masses in the marrow cavity and cortex **(A and B)**. Precontrast MRI showed the irregular mass was hypo-intense on T1WI **(C and E)** and hyper-intense on T2WI **(F)**. Contrast MRI showed the mass had significant enhancement **(D and G)**. **(H & E)** staining (H ×100) showed the tumor tissue consisted of poorly differentiated small round cells.

**Figure 8:**
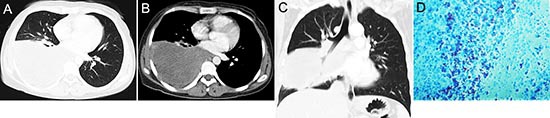
pPNET in a 30-year-old woman CT imaging showed an irregular iso-dense mass in the right lower lung **(A)** that invaded the mediastinum **(B)**. Coronal images using lung windows demonstrated invasion of the right lower lobe bronchus **(C)**. The poorly differentiated small round tumor cells were CD99 positive (D × 100).

The cut surface of pPNETs was gray white and granular. It had significant necrosis and vague boundaries between the tumor edge and normal tissue. Most (33/36) tumor margins contained local invasion with tumor cells. These gross pathological changes were consistent with the appearance on CT and MRI. Tumors were composed of atypical, small round-oval and spindle-shaped cells (Figure 1–8). Many cells demonstrated abnormal increased caryocinesia. Cells were arranged into nests or cords with rosettes (Homer-Wright rosettes) (27/36) (Figure 5H). Immunohistochemical analysis of the 36 tumors revealed 36 were positive for CD99 (Figure 6H), 33 were positive for NSE, 24 were positive for Vim and 17 were positive for S100.

## DISCUSSION

Primitive neuroectodermal tumor (PNET) is a rare malignant tumor originating from neuroectoderm. It consists of primitive undifferentiated small round cells and can be divided into central PNET (cPNET) and peripheral PNET (pPNET) tumors based on the tissue of origin [2]. The radiologic findings of pPNET are not well documented which makes the preoperative diagnosis difficult. Therefore, in this study, we seek to find out the characteristic imaging and clinical features of pPNET which could be used for the diagnosis.

### Clinical features

pPNET can be found in all age groups, but, is more common in young children. Khong et al. [6] and Ibarburen et al. [7] reported that the mean age of patients they treated was 8.0 and 15.8 years old, respectively. Dick et al. [8] reported younger affected children, with a mean age of 4.4 years old. The mean of our patients was 30.0 years old (range: 1–66 years). This difference in age may be related to differences in tumor location from the various studies. Xinchun Li [9] found that pPNET arising in the abdomen and pelvis occurred more frequently in adults. Huang J [10] found that patients with pPNET arising in the kidney had a mean age of 35.0 years old. Dick EA [8] reported that peripheral PNET occurred more frequently in women. In contrast, our patients had a male predominance (25/36). This is likely due to the heterogeneity in these reported series.

Patients generally presented with rapidly enlarging masses and symptoms related to tumor mass effects [3]. Out of 29 patients presented with intermittent pain, 4 had cough, 1 was incidentally found on physical examination and 2 had dizziness and fatigue.

### CT and MRI findings

pPNET often occur in the thoracopulmonary region (Askin tumor), retroperitonium, and extremities [11]. Uncommon locations include the thyroid [12], kidney [13], breast [14], nose [15], adrenal gland [16], prostate [17], head and neck [18], abdomen, pelvic soft tissues [9], and bone [19]. Our patients had tumors in bone (*n* = 19: limbs 11, vertebrae 4, craniofacial skeleton 4), soft tissue (*n* = 10: pelvis 2, retroperitoneum 2, groin 1, nasopharynx 1, chest wall 2, mediastinum 2), and organs (*n* = 7: parotid 1, pulmonary 4, spleen 1, liver 1). pPNETs occurred most frequently in the bones of our patients, which was not consistent with previous reports.

pPNET were generally large soft tissue masses (>5 cm on average) with ill-defined margins and exhibited aggressive local extension into normal tissue [9], [20], [21], [22]. In our series, the average diameter of pPNET was even bigger (8.1 cm) than literature reports. The tumors were often iso-density and had areas of necrosis on pre-contrast CT images. The tumors were iso-intense or hypo-intense on T1WI and had heterogeneous hyper-intensity on T2WI [4], [23]. Calcification was rare in pPNETs [20], occurring in less than 10% of tumors [7]. Tumors demonstrated heterogeneous enhancement on contrast-enhanced CT/T1WI [20], [24]. In our group most tumors (CT: 31/36; MRI: 14/16) had heterogeneous intensity with small necrosis. We also concluded the characteristics of pPNET, one unique characteristic of pPNET was that tumors were iso-intense on T1WI (14/16) and T2WI (9/16), and scarcely had calcification (2.8%) and hemorrhage (8.3%). Another unique characteristics of pPNET was that most tumors (CT: 34/36, MRI: 15/16) had heterogeneous enhancement, of which 25 cases on CT and 14 cases on MRI had significant enhancement. These were in accordance with literature reports.

pPNET is a locally invasive tumor, the pPNET grew aggressively, showing both an infiltrative and an expansive pattern [7], [21], [25]. Our CT and MRI findings also showed that thirty of our patients had poorly defined margins (83.3%) and displaced surrounding organs. This was accordance with pathologic findings that there were vague boundaries between the tumor edges and normal tissues which contain local invasive tumor cells. Patients with pPNET often developed distant metastases, but lymph node involvement was uncommon, being reported in only 3 of 17 cases (17.6%) [26]. In our study, none of the patients had distant metastasis and only 3 (8.3%) had lymph node metastasis at the time of diagnosis. The low incidence of distant metastasis may be because these patients were screened for localized disease prior to referral for surgery.

### Pathological findings

pPNET belongs to the family of small round cell tumors, originating from primitive undifferentiated neuroepithelial cells. The pathological features were first reported by Hart [1] and defined by the WHO in 1993 [27]. The most important histological criterion was that the tumor is composed of small round cells tightly arranged in cords, nests or clusters to form rosettes and pseudorosettes (Homer-Wright daisy-group). In our study, small round cells were observed in 36 tumors (100%) and Homer-Wright daisy-groups were found in 27 tumors (66.7%). Strong immunoreactivity for CD99 and neuronal markers such as NSE, and synaptophysin strongly support the diagnosis of pPNET [27]. NSE and CD99 were highly expressed in pPNET in most reports [28–30]. CD99 was reported to be expressed in most malignant pPNET and Ewing's sarcoma [20]. In our study, out of 36 patients, CD99 was expressed in all 36 patients, NSE was expressed in 33 patients, vimentin was expressed in 24 patients, and S100 was expressed in 17 patients. So demonstration of CD99 and NSE expression by immunocytochemical staining (CD99) aids in diagnosis pPNET.

### Treatment and prognosis

Surgery, radiation therapy and chemotherapy are the primary treatments for pPNET. The patient responses for these treatments were poor and had low survival rate. Patients with pPNET often developed distant metastases and local recurrence within 2–3 years after surgery. Distant metastases most often occurred in the lung, bone, liver, adrenal gland, brain, and retroperitoneum [11]. Jurgens et al. [31] reported a 5-year disease-free survival rate of 45–55%. Ibarbuan et al. [7] reported that the 3-year survival rate in pPNET patients receiving postoperative chemotherapy was 30%. In our study, the postoperative follow-up rate was 69.4% (25/36). Twelve patients died of which 9 had metastases and 3 had complications, 9 patients recurred of which 3 had metastases and 6 local recurrences, and 4 remained free of disease (12 months clinical follow up after surgery). The 1-year survival rate in pPNET patients receiving postoperative chemotherapy was 52%. We found that pPNET patients arising from organs had a much poorer prognosis than those tumors arising in bone or soft tissues.

In conclusion, the further diagnosis of pPNET should be suggested in young men when the following criteria was matched: images show a single large ill-defined solid mass with small area of necrosis; scarcely had classification or hemorrhage; locally invasive to adjacent structures, especially for those show iso-intense on T1WI and T2WI, which have significant enhancement.

## MATERIALS AND METHODS

### Clinical data

Fifty-three patients with pPNETs diagnosed by pathology were collected between 1 January 2006 and 1 December 2013 at two hospitals. Inclusion criteria: patients with pPNETs underwent a dynamic CT or MRI examination before surgery. Exclusion criteria: recurrent pPNETs and pPNETs treated with radiotherapy or chemotherapy before CT or MRI examination. According to the criteria, 36 patients underwent a dynamic CT examination and of which 16 underwent MRI before surgery. Thirty-three patients underwent tumor resection and 3 underwent tumor biopsy. The institutional review board (IRB) of our hospitals approved this retrospective study and informed consent was waived.

### Imaging

A Siemens Somatom Sensation multidetector 64-section CT system (Germany) was used for imaging. Settings were as follows: 3 mm section thickness, 5 mm intersection gap, field of view: 25 × 25 cm^2^–38 × 38 cm^2^, and matrix 512 × 512. Iohexol was used as the contrast agent at a dose of 1.5 ml/kg. The injection flow rate was 2.5 ml/s. A 25–30 s after the injection, arterial phase scanning was performed. Venous phase scanning was performed approximately 50 s after injection, and delayed phase scanning was performed 5 min after injection. The raw data were reformatted in the coronal and sagittal planes with a 3 mm section thickness and a 3 mm interval.

All examinations were performed with a Signa HDX 3.0 T MRI scanner using an eightchannel array coil. Settings were as follows: 6 mm section thickness, 1.5 mm intersection gap, field of view 380 × 380, and matrix 288 × 288. A respiratory triggered fast spin-echo (FSE) T1-weighted sequence [FSET1 with 250 ms repetition time (TR), 2.3 ms echo time (TE)] and a FSE T2-weighted, fat-suppressed (TSET2) sequence (2000 ms TR, 70 ms TE) were used. FSET1-enhanced MRI was performed using Gd-DTPA as the contrast agent. A dose of 0.2 mg/kg was used with an injection flow rate of 1.5 ml/s. Arterial phase imaging was performed approximately 25–30 s after injection, venous phase imaging approximately 50 s after injection, and delayed phase imaging approximately 5 min after injection.

### Imaging analysis

The tumor location, morphology, size, margin (well-defined or ill-defined) were measured. The signal intensity, CT density, lesion texture (homogeneous, or heterogeneous, or necrosis), contrast enhancement characteristics (homogeneous or heterogeneous; mild, moderate, significant), and involvement of surrounding soft tissue of the 36 tumors were also determined.

### Pathological examination

Thirty-three patients underwent tumor resection and 3 patients underwent biopsy. The histological analysis consisted of hematoxylin and eosin (HE) staining and immunohistochemical (IHC) staining. Neuron specific enolase (NSE), cluster of differentiation 99 (CD99), S100- protein, and vimentin (VIM) expression were examined. All the tumor specimens were reviewed by pathologist. The diagnosis of pPNET was made when there was evidence showing that cells were small round-oval and spindle-shape, which arranged with or without rosettes (Homer-Wright rosettes), and were positive for two or more kinds of neuron-specific antibodies.

### Statistical analysis

SPSS18.0. statistical analysis software was used. The age of patients with pPNET in different locations were compared by using one-way ANOVA test. *P* < 0.05 was considered to be statistically significant.
